# Isolation and identification of a male-produced aggregation-sex pheromone for the velvet longhorned beetle, *Trichoferus campestris*

**DOI:** 10.1038/s41598-019-41047-x

**Published:** 2019-03-14

**Authors:** Ann M. Ray, Joseph A. Francese, Yunfan Zou, Kristopher Watson, Damon J. Crook, Jocelyn G. Millar

**Affiliations:** 1 Department of Biology, Xavier University, 1548 Musketeer Drive, Cincinnati, OH 45207 USA; 2 USDA-APHIS-PPQ-CPHST Otis Laboratory, 1398 W. Truck Road, Buzzards Bay, MA 02542 USA; 3 Department of Entomology, University of California, 900 University Avenue, Riverside, CA 92521 USA; 4 Utah Department of Agriculture and Food, 350 North Redwood Road, PO Box 146500, Salt Lake City, Utah 84114 USA

## Abstract

The velvet longhorned beetle, *Trichoferus campestris* (Faldermann) (“VLB”; Coleoptera: Cerambycidae), is native to eastern Asia where it infests and damages a wide range of deciduous and coniferous tree species, including orchard and timber species. Immature stages of VLB are transported to new countries via international commerce, and populations have established outside the native range of the species. Here, we show that identification of pheromones of invasive pest species can be expedited by knowledge of the semiochemistry of related taxa. Histological sectioning revealed subcuticular, male-specific prothoracic glands connected to pits in the cuticle, which, in related species, are diagnostic for production of male-produced aggregation-sex pheromones, usually characterized by 2,3-alkanediol/hydroxyketone structural motifs. However, in preliminary field bioassays, beetles were not attracted by any known cerambycid pheromones. Subsequently, we identified a novel variant of the hydroxyketone motif (“trichoferone”) from headspace volatiles of males. In field bioassays, synthetic trichoferone was more attractive to both sexes of VLB than previously developed high-release-rate ethanol lures, and attraction was strongly female biased. This study demonstrated the utility of the prothoracic gland trait for predicting pheromone use in cerambycid species in the subfamily Cerambycinae, and that identification of pheromones of novel species can be expedited by knowledge of pheromones of related species. Trichoferone should prove to be a valuable tool for detection of VLB in regions where the beetle is or may become established.

## Introduction

Non-native woodboring insects pose a major threat to the health and biodiversity of natural and managed forest ecosystems worldwide^1–3^. Since the 1980s, establishment of exotic woodborers in North America has accelerated, likely correlated with increases in global trade, containerized shipping, and increased importation of wooden products from Asia^3–6^. Despite regulatory efforts that specify treatment of wood and inspection of high-risk cargo, live individuals of non-native woodborers are routinely intercepted at ports of entry^7^, and new outbreaks of potential pest species are detected every year^8^. Woodboring beetles in the family Cerambycidae present special challenges for regulatory personnel because the immature life stages feed inside dry wooden containers, pallets, and dunnage, and may escape detection^1^, complicating efforts to prevent introduction, establishment, and spread of exotic woodborers^9^.

Eradication programs for non-native, potential pest species are most successful when outbreaks are detected early and addressed quickly^10,11^. Volatile pheromones^12^ are widely used as tools for monitoring and control of insect pests, and traps baited with cerambycid pheromones have been used successfully in surveys for longhorned beetles in both managed and natural forests^13^. Because pheromone-baited traps are specific to target species, impacts on non-native species and costs of labor for sorting and identifying insects can be minimized. Moreover, pheromone-baited traps represent one of the most effective methods of detecting insect populations at the low densities that generally occur in the initial stages of a new introduction or outbreak^2,14–16^.

To maximize the possibility of eradicating exotic pests, it is critical that pheromones or other attractants be identified and deployed by regulatory agencies as soon as possible after the initial discovery of an incipient infestation^11^. In cerambycids, these efforts can be accelerated by exploiting what is known about the semiochemistry of related species^17,18^, particularly because pheromone structures are often conserved among related insect taxa. Similarly, the presence of morphological structures associated with pheromone production in related species can also inform efforts to identify pheromone structures. For example, males of many species in the subfamily Cerambycinae produce volatile aggregation-sex pheromones from subcuticular glands connected to pores on the prothorax^19^. Furthermore, the pheromones of species with pores usually consist of 2,3-alkanediols or hydroxyalkanones^20^. By examining prothoraces of males for pores, we can predict whether a species may use a pheromone of this motif, narrowing the search for male-specific compounds in extracts of headspace volatiles, and expediting identification of pheromones. The value of this “pheromone identification by proxy” strategy has previously been demonstrated in species with pheromones conforming to other structural motifs^17^.

The velvet longhorned beetle, *Trichoferus campestris* (Faldermann) (“VLB”; Cerambycidae: Cerambycinae: Hesperophanini), is native to northeastern Asia, where it attacks a wide range of forest, fruit, and shade tree species^21,22^, boring through vascular and structural tissues and weakening or killing its hosts^4,21,23,24^. In its native range, VLB is considered a serious pest in managed and natural forests, and of lumber and wooden structures^21,25^. Feeding by larvae of VLB may decrease plant and fruit yield in horticultural operations and commercial orchards^4^. The host range is not fully known, but data gathered to date suggest that VLB attacks trees in 40 genera, including both conifers and hardwoods^21,26^. The potential economic damage by VLB in North America is estimated to be high because of the broad host range of the beetle, and because VLB is a pest both in its native range and in areas where it has become established^24,27^.

All life stages of VLB are readily transported in wooden products and packing materials^5,7,27^, and VLB has established in many locations outside of its native range. Currently, VLB is a quarantine species in both the U.S. and Canada^28,29^. It is known to be established throughout the Middle East and Europe, as well as in parts of South and Central America, and its range is expected to expand further^5,24,30–32^. In the U.S., VLB are commonly intercepted in shipments originating from northeastern Asia, although in recent years live individuals of VLB have also been intercepted in shipments originating outside of the species’ native range (i.e., Brazil, Italy, Mexico, Spain^7,27^).

The first discovery of VLB adults in North America (outside of a quarantine facility) was in Repentigny, Quebec, Canada in 2002^33^. Additional populations of VLB have since been discovered near Chicago, Illinois, and in four counties near Salt Lake City, Utah^8,26^. Small numbers of VLB adults have also been collected during surveys throughout the U.S. and Canada^8,34^, suggesting that incipient populations of this species may be present in a number of areas throughout North America.

The life history parameters of VLB in North America are practically unknown^9,33^. The literature contains conflicting data regarding the ability of VLB to infest live trees, rather than stressed, moribund, or dead trees^9,22,35^. As this invasive species continues to expand its range, more detailed information as to its host range and potential for economic damage, and better methods of detection and monitoring its populations, are urgently required^2,27^. As an important first step to meeting these requirements, we describe here the identification, synthesis, and field testing of a male-produced aggregation-sex pheromone from VLB. We anticipate that this compound will be a valuable tool for management in orchards, managed and natural forests, and ornamental plantings.

## Results

### Location of pheromone-producing glands and pores

**Figure 1:**
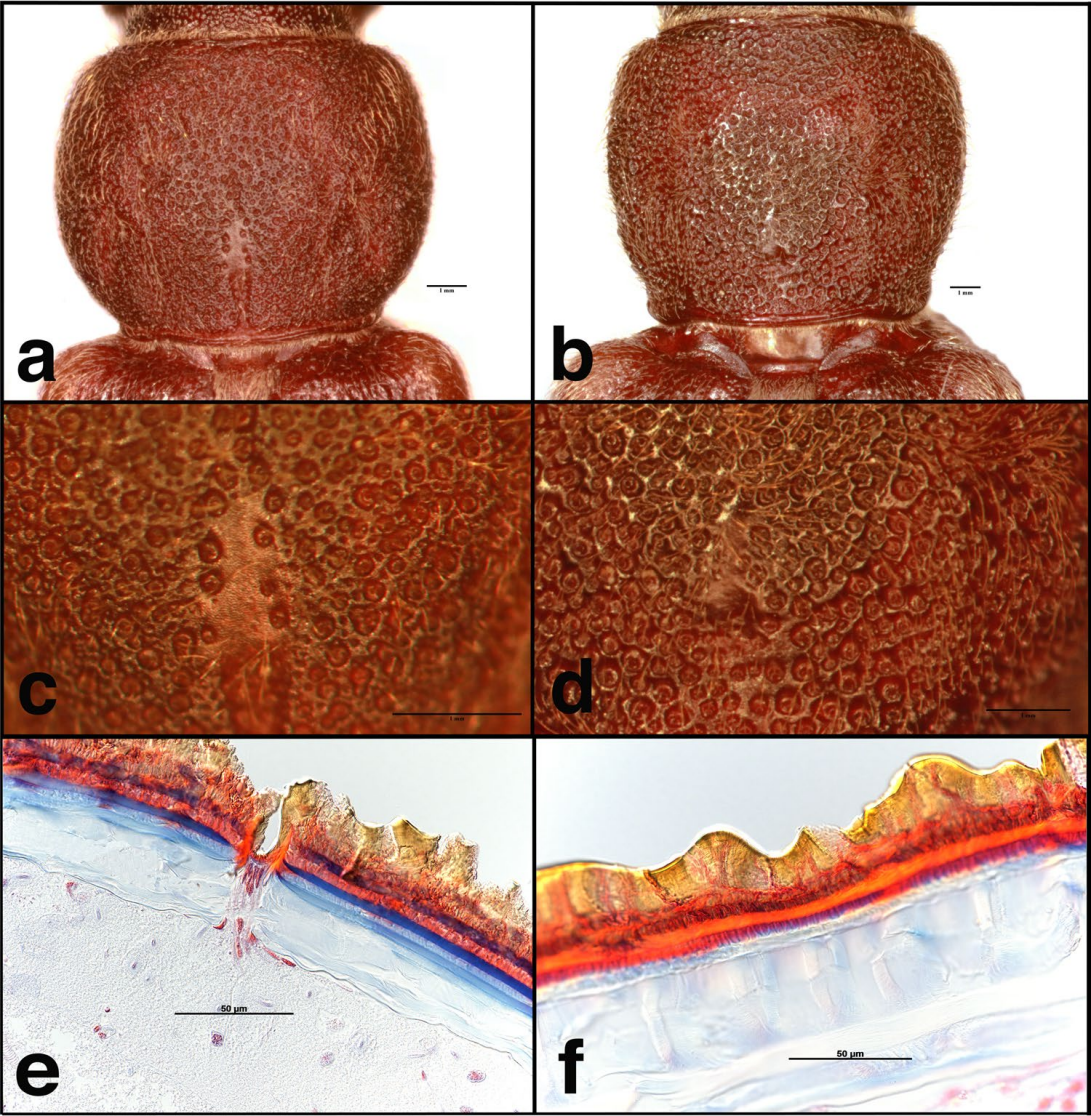
Images of the prothoraces of adult male (**a**) 20X magnification, (**c**) 60X magnification, and (**e**) 40X magnification, cross section and female (**b**) 15X magnification, (**d**) 40X magnification, and (**f**) 40X magnification, cross section *T*. *campestris* showing sexual dimorphism in shape, punctuation, and presence of subcuticular glands.

As reported previously, prothoraces of adult male and female VLB are sexually dimorphic (Fig. 1a,b)^33^. The prothoracic cuticle of males is more densely punctate than that of females (Fig. 1c,d). Histological sectioning revealed that the pores in the prothoraces of males are associated with subcuticular glands (Fig. 1e, Supplementary Fig. 1). Females lack both the pores and the associated glands (Fig. 1f, Supplementary Fig. 1). These glands are associated with the production of male-specific aggregation-sex pheromones in other species in the cerambycid subfamily Cerambycinae, including other species in the tribe Hesperophanini (e.g., *Brothylus gemmulatus* LeConte)^19,36,37^.

### Identification of the *T*. *campestris* pheromone

Analyses of extracts of headspace volatiles from both sexes of *T*. *campestris* revealed the presence of two apparently isomeric, male-specific compounds in some extracts from males. The EI mass spectrum of the major component had a prominent ion at 188 and a trace ion at *m/z* 206, likely the molecular ion, with a base peak at *m*/*z* 121. It was not possible to get an exact mass measurement on the molecular ion, but the *m*/*z* 188 ion was determined to have the formula C_13_H_16_O (calc. 188.1196; measured 188.1205), and so the molecular ion at *m*/*z* 206 thus corresponded to C_13_H_18_O_2_, with 5 sites of unsaturation. The compound was not reduced by catalytic hydrogenation with palladium on carbon, indicating that there were no C-C double or triple bonds present. However, the molecule did react with LiAlH_4_ in ether, with the *m*/*z* 188 ion increasing to 190 in the mass spectrum of the reduced product, indicating the presence of one carbonyl that was not an ester.

The microprobe proton NMR spectrum of the major component showed two multiplets at 7.32 and 7.23 ppm corresponding to a total of 5 protons, indicating a monosubstituted benzene ring with no strongly electron-withdrawing or donating groups directly attached to the ring, and accounting for 4 of the 5 sites of unsaturation. A distorted triplet at 0.88 ppm (J = 6.9 Hz) integrating to 3 protons corresponded to an alkyl methyl group attached to two diastereotopic methylene protons at 1.67 (dqd, J = 14.1, 6.8, 6.7 Hz) and 1.44 ppm (dqd, J = 14.1, 6.9, 6.8 Hz) respectively. On the other side, these were coupled to a one-proton multiplet at 2.75 ppm, which in turn was further coupled to a methyl group at 1.05 ppm (d, J = 6.7 Hz). The downfield shifts of the methine and the methyl suggested that they were adjacent to a carbonyl group. The remaining four protons consisted of a diastereotopic methylene group at 3.15 ppm (dd, J = 14.1, 4.1 Hz) and 2.75 ppm (likely dd, J = 14.1 Hz, second coupling not discernable because of overlap with methine described above), which was coupled to a methine at 4.48 ppm (m), which in turn was coupled to a proton at 3.37 ppm (d, J = 5.8 Hz). The chemical shifts and couplings suggested a spin system consisting of a benzylic methylene and a secondary alcohol, which was adjacent to the carbonyl. Because gCOSY spectra did not show up several of the expected couplings, several multiplets were individually decoupled. Irradiation of the methyl triplet at 0.88 ppm collapsed the diastereotopic methylenes at 1.67 and 1.44 ppm to doublets of doublets. Irradiation of the multiplet at 2.75 ppm, corresponding to one of the benzylic protons and the methine coupled to the methyl group at 1.05 ppm, collapsed the proton at 4.48 ppm to a dd, the methyl group to a singlet, and the two diastereotopic protons at 1.67 and 1.44 ppm to dqs. In sum, these data suggested the gross structure 2 (Fig. 3), which was confirmed by synthesis of a mixture of all 4 isomers, followed by several stereoselective syntheses to determine which isomers the insect actually produced.

### Synthesis of the putative *T*. *campestris* pheromone as a mixture of stereoisomers and determination of the stereochemistry of the insect-produced compounds

**Figure 2:**
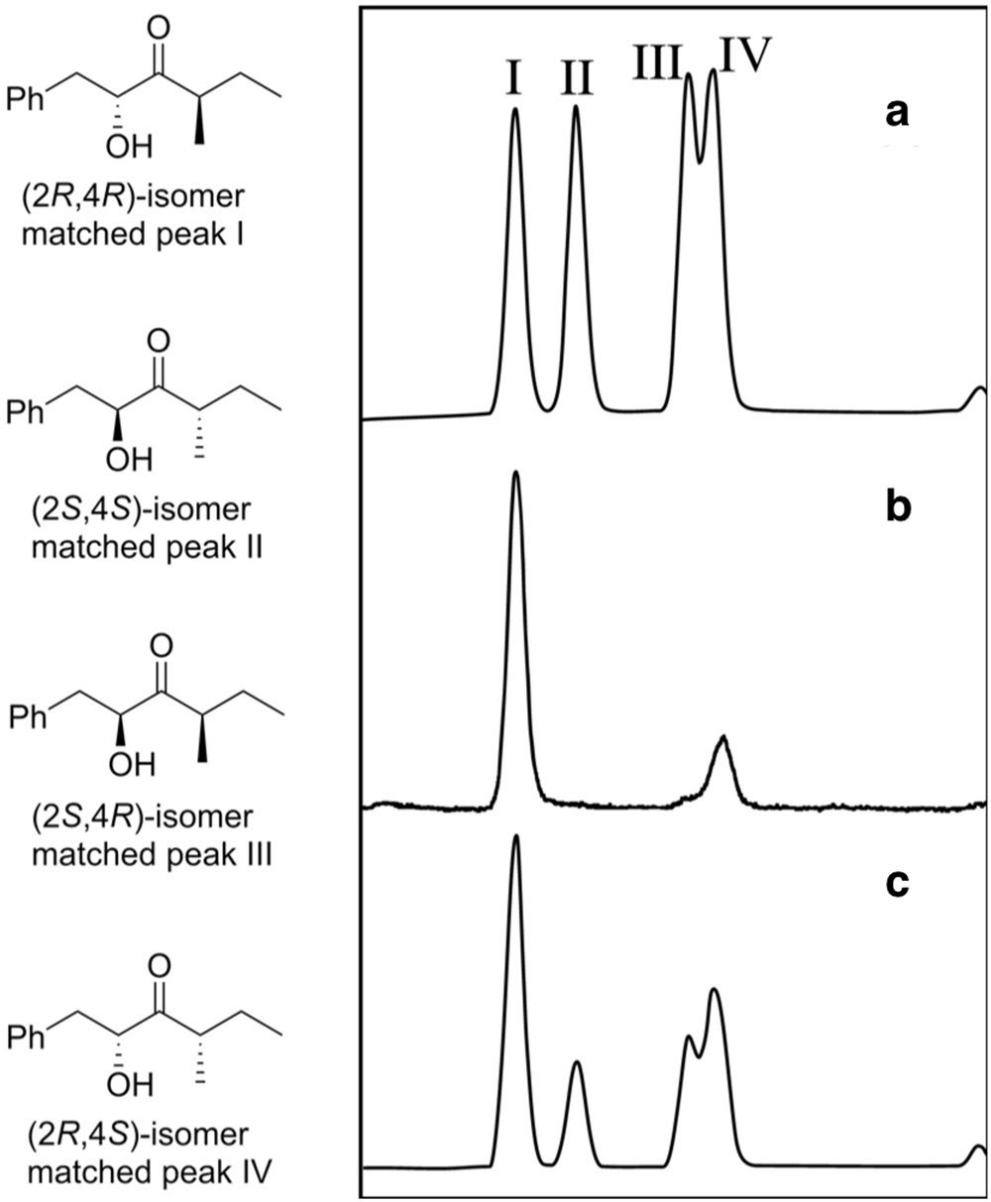
Structures of stereoisomers of 2-hydroxy-4-methyl-1-phenylhexan-3-one (left) and gas chromatograms on a chiral stationary phase Cyclodex B column (right). (**a**) All four stereoisomers of 2-hydroxy-4-methyl-1-phenylhexan-3-one; (**b**) insect-produced compounds; (**c**) co-injection of all 4 isomers with the insect extract, showing that peaks I and IV are enhanced.

**Figure 3:**
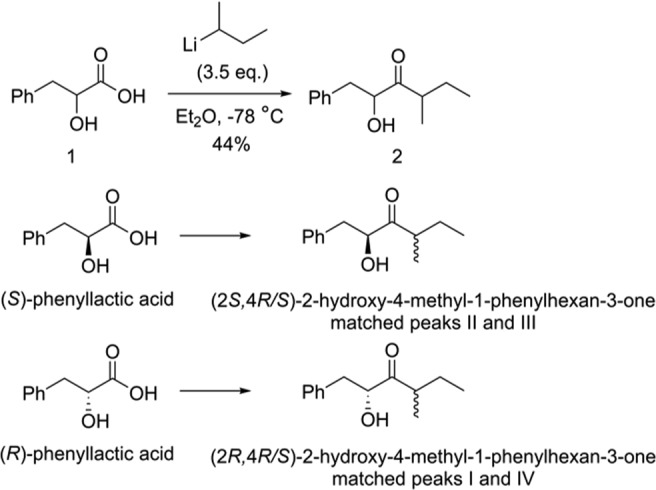
Synthesis of 2-hydroxy-4-methyl-1-phenylhexan-3-one (**2**) as a mixture of 4 stereoisomers. The reaction was repeated using (*S*)- or (*R*)-phenyllactic acids as starting materials, to give (2*S*,4*R/S*)-2-hydroxy-4-methyl-1-phenylhexan-3-one and (2*R*,4*R/S*)-2-hydroxy-4-methyl-1-phenylhexan-3-one respectively.

Our synthesis was based on a modification of work by Aronoff *et al*.^38^, who recently reported a synthesis of α-hydroxyketones analogous to target compound **2**, which did not require protecting groups. They reported that reaction of the Weinreb amide of (*S*)-phenyllactic acid with 2.5 equivalents of isobutyl lithium or *n*-butyllithium at low temperature gave α-hydroxyketones cleanly in excellent yield^38,39^. However, when the same reaction conditions were applied to the Weinreb amide from DL-phenyllactic acid **1** and *sec*-BuLi, the reaction was messy and the desired product was obtained in poor yield (18%). However, in our hands, we found that treatment of DL-phenyllactic acid **1** directly with 3.5 equiv of *sec*-BuLi gave a much cleaner reaction (Fig. 3). The choice of solvent (Et_2_O vs. THF) and temperature (−78 °C vs. 0 °C) was critical. Under optimized conditions (Et_2_O, −78 °C), the desired product **2** was obtained in 44% yield in one step, as an approximately equal mixture of the four stereoisomers. The stereoisomers I–IV were adequately resolved on a Cyclodex B chiral stationary phase GC column (Fig. 2a), although the relative and absolute stereochemistries of each were unknown at this point.

**Figure 4:**
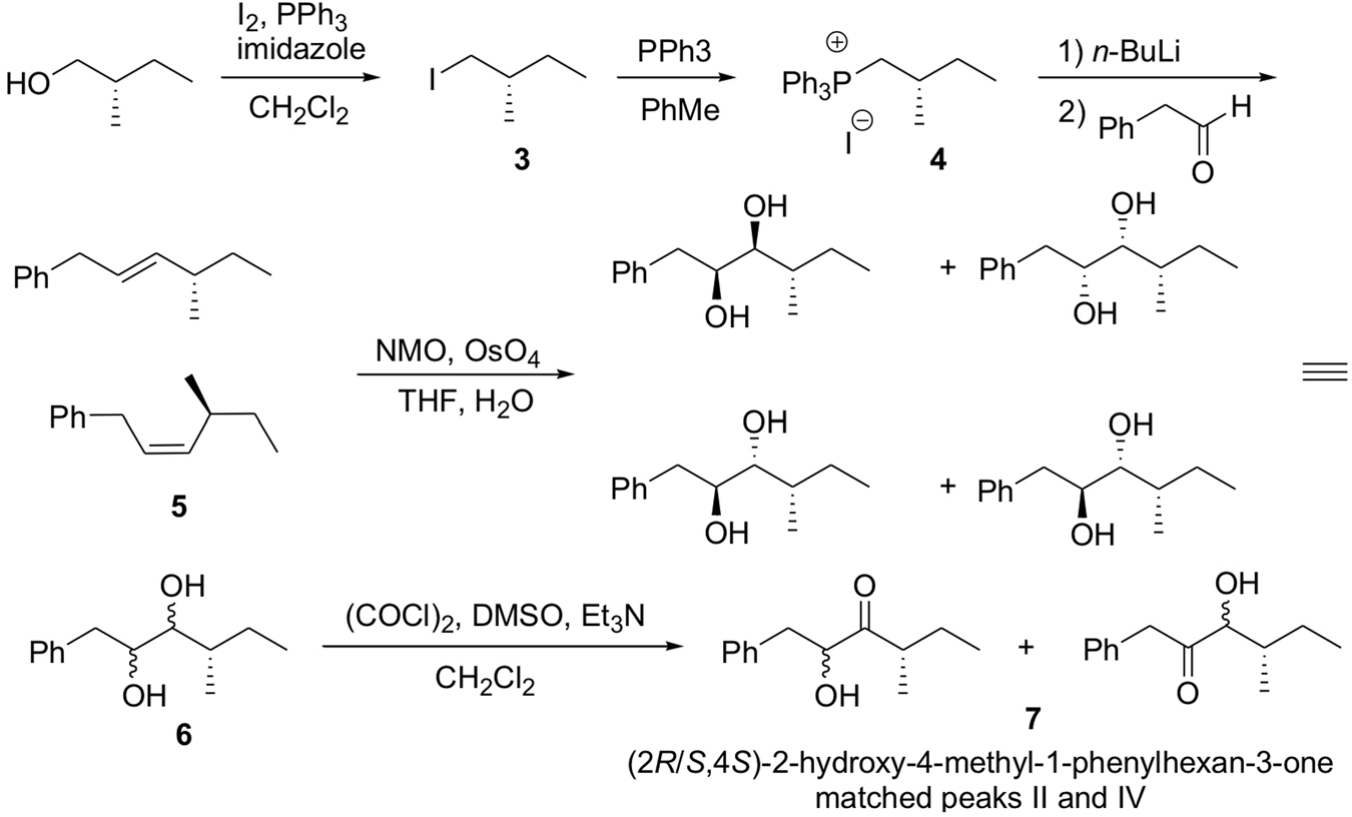
Synthesis of (2*R*/*S*,4*S*)-2-hydroxy-4-methyl-1-phenylhexan-3-one.

Both enantiomers of the phenyllactic acid starting material were commercially available. Under the same reaction conditions, (2*S*,4*R/S*)-2-hydroxy-4-methyl-1-phenylhexan-3-one was prepared from (*S*)-phenyllactic acid, and (2*R*,4*R/S*)-2-hydroxy-4-methyl-1-phenylhexan-3-one was prepared from (*R*)-phenyllactic acid (Fig. 3). The two diastereomers of (2*S*,4*R/S*)-2-hydroxy-4-methyl-1-phenylhexan-3-one matched peaks II and III in the chiral column GC analysis, and those from (2*R*,4*R/S*)-2-hydroxy-4-methyl-1-phenylhexan-3-one matched peaks I and IV. To finish assignment of the stereochemistries of all the isomers, we then prepared a sample of the two diastereomers with the stereochemistry at position 4 fixed as (*S*) (Fig. 4). On chiral GC, the diastereomers matched peaks II and IV, identifying II as the (2*S*,4*S*)-isomer, and IV as the (2*R*,4*S*)-isomer. Then, knowing that peak I had the (*R*)-configuration at carbon 2 and that III had the (*S*)-configuration at carbon 2, by process of elimination, this unequivocally identified I as (2*R*,4*R*) and III as (2*S*,4*R*) (Fig. 2).

Finally, analysis of the insect extract under the same conditions showed that the insects produced a mixture of I (major isomer, (2*R*,4*R*)) and IV (2*R*,4*S*) (Fig. 2b,c).

### Field bioassays

In preliminary field bioassays in 2014 and 2015, we tested a variety of known semiochemical attractants for other species in the subfamily Cerambycinae, including 3-hydroxydecan-2-one, 2-hydroxy-4-octen-3-one, *syn*-2,3-octanediol, *anti*-2,3-octanediol, 3-hydroxyoctan-2-one, *syn*-2,3-hexanediol, *anti*-2,3-hexanediol, 3-hydroxyhexan-2-one, 3-hydroxybutan-2-one, and Ultra High Release [UHR] ethanol (Supplementary Table S1). However, none of the treatments were significantly more attractive than isopropanol solvent or blank controls (Supplementary Figs S2 and S3). This suggested that the pheromone of VLB was likely a novel compound, so we proceeded with collection and analysis of beetle-produced volatiles, as described above.

We captured a total of 3,014 VLB in the two bioassays in 2016. Catch was strongly biased toward females in all traps (all treatments and controls) (Table 1). Significantly more VLB were caught in traps baited with trichoferone than in UHR ethanol traps, solvent, or blank control traps (Table 1). There was no significant difference in attraction between the blend of four stereoisomers and the blend of the major insect-produced (2*R*,4*R*)-stereoisomer and the (2*R*,4*S*)-diastereomer. Traps baited with the blend of four stereoisomers alone and in combination with UHR ethanol lures captured significantly more beetles than did traps baited with UHR ethanol alone, solvent controls, or blank controls (Table 1).

**Table 1:** Mean (±s.e.m.) number of beetles captured per trap per week during field bioassays in 2016. Sex ratio (% females of total) is from beetles captured across all treatments.

Dates	# of obs. (N)	Total # beetles	Sex ratio (% female)	Treatments						
				*syn*-/*anti-*isomers	(2*R*,4*R*/*S*)-isomers	*syn*-/*anti-*isomers + UHR ethanol	UHR ethanol	solvent	blank	Χ^2^ ANOVA
21-VI—19-VII	40	2,257	70.8	21.3 ± 2.6 **a**	16.7 ± 1.7 **a**		9.8 ± 1.2 **b**	4.5 ± 0.94 **c**	4.18 ± 0.80 **c**	89.99
19-VII—2-VIII	20	751	78.0	15.8 ± 3.0 **a**		14.4 ± 2.3 **a**	4.6 ± 1.1 **b**	2.1 ± 0.67 **b**	1.15 ± 0.26 **b**	54.60

Empty cells indicate treatments that were not included in a particular bioassay. Significantly more VLB were captured in traps containing trichoferone alone and in combination with UHR ethanol than in solvent (isopropyl alcohol) or blank controls. The addition of UHR ethanol did not synergize attraction to traps (Friedman’s ANOVA, test statistics above, p > 0.00001). Means with the same letters are not significantly different at α = 0.05.

A total of 583 adults of nine other species of cerambycids were captured in traps in bioassays during 2016. Only 28 individuals of any species were captured in traps baited with pheromone treatments, and there was no observable treatment effect for most species (raw data available at www.exhibit.xavier.edu/biology_faculty/107). However, 524 *Neoclytus a*. *acuminatus* F. were caught, with 85% of individuals captured in traps baited with ethanol, a known attractant for some cerambycids^40^.

## Discussion

The experiments described here indicate that male VLB produce a sex-specific aggregation-sex pheromone (*sensu* Cardé)^12^ comprising two isomers of 2-hydroxy-4-methyl-1-phenylhexan-3-one in an approximately 3:1 ratio. To our knowledge, these compounds have not previously been identified as semiochemicals from any other animal species^41^, and thus trichoferone represents a novel variant of the 2,3-hydroxyketone structures that are common pheromone components for species in the subfamily Cerambycinae^18^. In addition, no other cerambycid species were significantly attracted to trichoferone at any of our field sites. There are no native North American *Trichoferus* species, but there are a number of *Trichoferus* species known from Europe and Asia^42^. It remains to be determined whether trichoferone may be attractive, and hence a likely pheromone component, for one or more of these congeners.

Our results also show how knowledge of the pheromone chemistry and biology of related species can expedite the identification of novel pheromone structures. In particular, from the taxonomic placement of VLB in the subfamily Cerambycinae and the tribe Hesperophanini, we hypothesized that pheromones would likely be produced by adult males and might conform to the well-known hydroxyketone or 2,3-alkanediol structural motifs, consistent with many other species in the same subfamily and tribe^37,43^. The presence of male-specific prothoracic pores and glands was also consistent with a likely male-produced pheromone^19,37^. However, the failure of VLB adults to respond to a panel of pheromones known from other members of the Hesperophanini^18^ during field testing suggested that the pheromone might be new. The pheromone components were indeed found to be novel, but they still contained the characteristic vicinal hydroxyketone motif common to pheromones of many members of the subfamily^18^.

In field bioassays, we determined that VLB were equally attracted to lures containing a blend of all four stereoisomers of trichoferone, and to lures containing only the mixture of the (2*R*,4*R/S*) diastereomers (Table 1), suggesting that the presence of “non-natural” stereoisomers does not inhibit response of beetles to traps, as observed in some other cerambycine species^44,45^. To date, given the attraction to both the 4-component and 2-component blends, we have not synthesized and field tested the (2*R*,4*R*)- and (2*R*,4*S*)-stereoisomers, for example, to test whether the two naturally produced stereoisomers act additively or synergistically. This pheromone may represent one of the rare instances in which the more stereoisomerically pure synthetic pheromone, i.e., the 2-component blend, is actually less expensive to produce than the racemate because of the relative costs of the enantiomerically pure starting material versus the corresponding racemate.

In subsequent field bioassays, we found no evidence that ethanol, a possible host plant volatile, enhanced attraction of beetles to pheromone-baited traps (Table 1) as has been reported for some other cerambycids^44,46^. Further research may be warranted to explore possible additive or synergistic effects of other plant volatiles, particularly once feeding and oviposition preferences of VLB are more clearly delineated.

An important step in assessing the potential economic impact of VLB is determining its current distribution and rate of range expansion. The identification of trichoferone as an attractant should provide a sensitive and species-specific tool for detecting and monitoring VLB. Trichoferone-baited traps have already demonstrated their effectiveness at detecting VLB at low population densities. Specifically, traps baited with trichoferone captured VLB adults in two U.S. states where populations were not known to exist (Ohio and Pennsylvania; JAF and AMR pers. obs., S. Spichiger pers. comm.)^8^. Early detection is critical for successful management of invasive species^3^, and eradication efforts are most successful when populations are still small and localized^11^. Although it may be too late to eradicate VLB from North America, pheromone-baited surveillance traps may still form a first line of defense in slowing its spread to other countries and continents.

## Methods

### Location of pheromone-producing glands and pores

Ten beetles of each sex, collected in traps during field bioassays in 2014, were examined using an Olympus SZX7 stereomicroscope to assess presence/absence of pores in males and females. Images were taken using a Canon EOS 70D camera attached to a light microscope digital SLR adapter on a Nikon SMZ1000 stereomicroscope equipped with a Plan Apo 1X WD70 objective lens (8–80x total magnification).

Specimens for histological sectioning were collected in live traps (described below), placed adjacent to the Adams Produce site (see below for details about field site). Specimens were submerged in ethanol-based hand sanitizer as a fixative. Fixation, sectioning, and imaging were contracted to Laudier Histology (New York, NY). Images were acquired from microscope slides with a Carl Zeiss AxioImager Z1 microscope, running ZEN Blue image processing software (Zeiss, Oberkochen, Germany).

### Collection of headspace volatiles

Eleven live *T*. *campestris* adults (7 females, 4 males) were collected between 13–16 July 2015, from traps placed at the Adams Produce site (see below for details about field site). Live trapping was conducted using five black intercept panel traps (Alpha Scents Inc., West Linn, OR) coated with Fluon® PTFE by the trap manufacturer, placed ~1.5 m above the ground and fitted with dry collecting cups, and two blacklight bucket traps. Intercept traps were baited with UHR ethanol lures (0.5 g/d, Alpha Scents Inc., West Linn, OR). We checked all traps daily, at which time beetles were placed individually into containers with moistened dental wicks to prevent dehydration during transport to the laboratory of the United States Department of Agriculture-Animal Plant Health Inspection Service (USDA-APHIS; West Valley, UT).

Headspace volatiles were collected from individual beetles. Depending on the number of beetles available, at least one control collection was performed with an empty jar during every sampling period. For collection of volatiles, air was pulled through a 360 mL PFA jar containing a beetle at 0.5 L/min with Portable Volatile Assay System pumps (Volatile Assay Systems, Rensselear, NY). Each jar was fitted with an 89 mm transfer closure, in turn fitted with two 0.64 cm outer diameter tube ports for making connections (Savillex, Eden Prairie, MN). Incoming air was filtered through a 400 mg charcoal filter (Orbo 32 large, Sigma Aldrich, Milwaukee, WI). Volatiles were collected with a 10.2 cm glass tube (0.4 cm ID × 0.6 cm OD at the air intake tapered to 0.2 cm ID × 0.4 cm OD at the jar inlet) filled with 20 mg of HayeSepQ® adsorbent by the manufacturer (Volatile Collection Traps LLC, Gainesville, FL).

Volatiles were collected under ambient environmental conditions at the USDA-APHIS laboratory (West Valley, UT) for 13–18 h, beginning between 17:00 and 18:00 and ending between 07:00 and 11:00 the next morning, during the nights of 13–16 July 2015 (VLB are nocturnally active; AMR and JAF, pers. obs.). At the end of the collection period, beetles were removed from jars and returned to individual containers with moistened dental wicks, then placed in a ~4 °C refrigerator during daylight hours to prolong the lives of the beetles. A total of 29 collections (6 from males, 16 from females, 7 from empty controls) were made over the 4-night period. The collectors then were shipped to University of California, Riverside for extraction and analysis.

Using the same method, volatiles were also collected from a single male adult VLB that was reared from a laboratory colony at the USDA-APHIS Otis Laboratory (Buzzards Bay, MA). Aerations were performed over four nights between 18–22 December 2015 from 15:00 to 07:00 the next morning. During each night, a collection was also performed with an empty jar as a control. Trapped volatiles were extracted with 1 ml of hexane, and concentrated under a stream of nitrogen to ~25 μl. Headspace extracts were stored at −18 °C until shipped to University of California, Riverside for analysis.

### Isolation and identification of the major male-produced compound

Extracts of volatiles were analysed by coupled gas chromatography-mass spectrometry (GC-MS) using an Agilent 7820 A GC (Agilent, Santa Clara, CA) coupled to a 5977E mass selective detector. The GC was fitted with a DB-5 column (30 m × 0.25 mm diameter, 0.25 μm film thickness; J&W Scientific, Folsom CA), programmed from 40 °C for 1 min, 10 °C min^−1^ to 280 °C, using helium carrier gas. Injections were made in splitless mode, with an injector temperature of 250 °C, and transfer line temperature of 280 °C. Mass spectra were taken in electron impact ionization (EI) mode at 70 eV. Exact masses were determined with a Waters GCT instrument in EI mode (70 eV).

An aliquot of an extract from male beetles was catalytically reduced by addition of ~1 mg 5% Pd on carbon, and stirring for 2 h under H_2_. After filtration through Celite, the resulting product was analysed by GC-MS. A second aliquot was concentrated just to dryness, then treated with 200 µl of a solution of LiAlH_4_ in ether (5 mg/ml) for 2 h at room temperature. The resulting mixture was diluted with ether, then carefully quenched with dilute aqueous HCl and vortexed. After separation of the layers, the ether layer was washed with saturated aqueous NaHCO_3_ solution and brine, dried over anhydrous Na_2_SO_4_, and analysed by GC-MS.

The two extracts that contained the largest amounts of the major-male specific compound were combined, concentrated, and purified by preparative GC on a Hewlett-Packard (now Agilent) 5890 GC equipped with a 25 m × 0.53 mm ID DB-5 column (5 micron film thickness), programming from 45 °C for 0 min, 10 °C min^−1^ to 260 °C, hold for 20 min, using helium carrier gas. The column effluent was split ~1:30 between the FID and a heated outlet port, with fractions being collected in dry ice cooled glass capillary tubes. After warming to room temperature, compounds were rinsed from the tubes with 25 µl of CD_2_Cl_2_, and a sample was transferred directly to a 1 mm ID microbore NMR tube. NMR spectra were taken using a Bruker Avance instrument at 600 MHz.

### Synthesis

Reactions using water- or air-sensitive reagents were carried out in oven-dried glassware, under argon atmosphere. Tetrahydrofuran was freshly distilled from benzophenone-sodium ketyl before use. Solutions were dried over anhydrous Na_2_SO_4_ and concentrated by rotary evaporation under reduced pressure. NMR spectra were taken on a Varian Inova 400 instrument, as CDCl_3_ or CD_2_Cl_2_ solutions.

### Synthesis of 2-hydroxy-4-methyl-1-phenylhexan-3-one (2) as a mixture of 4 stereoisomers

*Sec*-BuLi (1.4 M in cyclohexane, 50 mL, 70 mmol) (Fig. 3) was added dropwise to a solution of DL-phenyllactic acid **1** (3.32 g, 20 mmol) in Et_2_O (100 mL) cooled to −78 °C. After 30 min, the cold bath was removed and the reaction was warmed to room temperature. After 2 h the reaction mixture was poured into ice-water and extracted with Et_2_O. The combined organic layer was washed with saturated aqueous NH_4_Cl and brine, dried, and concentrated. The crude product was purified by flash chromatography (hexane/EtOAc = 9/1) to give **2** as an approximately equal mixture of 4 stereoisomers (yellow liquid, 1.82 g, 44%). ^1^H NMR (CD_2_Cl_2_, 400 MHz) δ 7.25–7.34 (m, 5H), 4.51 (ddd, *J* = 9.6, 8.4, 4.0 Hz, 1H), 3.15 (dd, *J* = 14.0, 4.0 Hz, 1H), 2.68–2.79 (m, 2H), 1.69 (m, 1H), 1.43 (m, 1H), 1.12 and 1.06 (d, *J* = 6.8 Hz, total 3H), 0.90 and 0.87 (t, *J* = 7.2 Hz, total 3H); ^13^C NMR (CD_2_Cl_2_, 100.5 MHz) δ 215.96, 215.87, 138.03, 137.87, 77.35, 76.38, 44.06, 43.61, 40.57, 27.72, 25.49, 17.68, 15.13, 12.13, 11.73; MS (*m*/*z*, rel. abundance) 41 (23), 45 (16), 57 (58), 65 (19), 77 (22), 79 (10), 85 (13), 91 (85), 92 (61), 103 (56), 120 (39), 121 (100), 131 (11), 188 (26).

The reaction was repeated using (*S*)- or (*R*)-phenyllactic acids as starting materials, to give (2*S*,4*R/S*)-2-hydroxy-4-methyl-1-phenylhexan-3-one and (2*R*,4*R/S*)-2-hydroxy-4-methyl-1-phenylhexan-3-one respectively (Fig. 2).

### Synthesis of (2*R*/*S*,4*S*)-2-hydroxy-4-methyl-1-phenylhexan-3-one

(*S*)-1-Iodo-2-methylbutane (**3**) Imidazole (2.04 g, 30 mmol) was added to a solution of PPh_3_ (6.29 g, 24 mmol) in CH_2_Cl_2_ (30 mL) at 0 °C followed by I_2_ (6.85 g, 27 mmol) **(**Fig. 4**)**. (*S*)-(−)-2-methyl-1-butanol (2.2 mL, 20 mmol) in CH_2_Cl_2_ (10 mL) was added slowly to the resulting slurry, and the mixture was stirred for 2 h while warming to room temperature. The reaction mixture then was poured into saturated Na_2_S_2_O_3_, and extracted with CH_2_Cl_2_. The organic layer was washed with brine, dried, and concentrated. Pentane was added to the residue to precipitate the bulk of the triphenylphosphine oxide, and the resulting mixture was filtered through Celite. The crude product was purified by vacuum flash chromatography (pentane) to give **3** as a light yellow liquid (1.70 g, 43%). ^1^H NMR (CDCl_3_, 400 MHz) δ 3.23 (dd, *J* = 9.6, 4.4 Hz, 1H), 3.17 (dd, *J* = 9.6, 6.0 Hz, 1H), 1.35–1.47 (m, 2H), 1.22–1.32 (m, 1H), 0.98 (d, *J* = 6.4 Hz, 3H), 0.89 (t, *J* = 7.2 Hz, 3H); MS (*m*/*z*, rel. abundance): 41 (52), 42 (15), 43 (97), 55 (23), 71 (100), 198 (M^+^, 20).

(*S*)-(2-Methylbutyl)triphenylphosphonium iodide (**4**) A mixture of iodide **3** (0.99 g, 5.0 mmol), and Ph_3_P (1.97 g, 7.5 mmol) in toluene (10 mL) was refluxed under argon for 2 d. The reaction mixture was cooled to room temperature, and the phosphonium salt was collected on a filter funnel, washed with Et_2_O and dried at room temperature overnight to give 4 as a white solid (1.37 g, 60%).

(*S*)-(4-Methylhex-2-enyl)-benzene (**5**) *n*-BuLi (2.2 M, 1.1 mL, 2.5 mmol) was added to a suspension of phosphonium salt **4** (1.15 g, 2.5 mmol) in THF (8 mL) cooled at 0 °C, and the mixture was stirred for 30 min. Phenylacetaldehyde (0.39 g, 3.25 mmol) in THF (2 mL) was added slowly, and the mixture then was stirred for 1 h before being poured into saturated aqueous NH_4_Cl, and extracted with hexanes. The organic layer was washed with water and brine, dried, and concentrated. The crude product was purified by vacuum flash chromatography (hexane) to give **5** as a colorless liquid (0.18 g, 41%). MS (*m*/*z*, rel. abundance) 41 (15), 55 (29), 65 (15), 77 (11), 82 (13), 83 (35), 91 (61), 103 (10), 104 (83), 105 (16), 115 (34), 117 (100), 118 (19), 128 (17), 129 (17), 130 (12), 145 (31), 174 (M^+^, 25).

(4*S*)-4-Methyl-1-phenylhexane-2,3-diol (**6**) OsO_4_ (2.5 wt% in *tert*-BuOH, 75 μL) and N-methylmorpholine N-oxide (50% w/w in H_2_O, 0.62 mL, 3.0 mmol) were added to a mixture of **5** (174 mg, 1.0 mmol) in THF (3 mL) and H_2_O (0.5 mL). The mixture was stirred vigorously at room temperature for 3 d, poured into saturated Na_2_S_2_O_3_ and stirred for 1.5 h, and extracted with EtOAc. The combined organic layer was washed with brine, 6 M H_2_SO_4_, saturated NaHCO_3_, and brine, then dried and concentrated. The crude product was purified by vacuum flash chromatography (hexane/EtOAc = 9/1 to 2/1) to give **6** as a colorless oil (148 mg, 71%). MS (*m*/*z*, rel. abundance) 41 (13), 43 (13), 45 (21), 57 (10), 65 (12), 69 (10), 77 (12), 78 (16), 87 (12), 91 (58), 92 (100), 93 (10), 103 (22), 104 (14), 121 (25), 122 (17).

(2*R*/*S*,4*S*)-2-Hydroxy-4-methyl-1-phenylhexan-3-one and (3*R*/*S*,4*S*)-3-hydroxy-4-methyl-1-phenylhexan-2-one (**7**) A solution of oxalyl chloride (51 μL, 0.60 mmol) in anhydrous CH_2_Cl_2_ (2 mL) was cooled to −78 °C under Ar and a solution of dimethylsulfoxide (89 μL, 1.25 mmol) in anhydrous CH_2_Cl_2_ (1 mL) was added dropwise. After 30 min, a solution of diol **6** (104 mg, 0.50 mmol) in anhydrous CH_2_Cl_2_ (1.4 mL) was added and the mixture was stirred for 1 h. Et_3_N (0.35 mL, 2.5 mmol) then was added and stirring continued for 30 min. The cold bath was removed and the mixture was stirred for 1.5 h while warming to room temperature. The mixture was poured into water and extracted with Et_2_O. The combined organic layer was washed with 1 M HCl, saturated NaHCO_3_, and brine, then dried and concentrated to give crude mixture 7, which was analyzed on a chiral stationary phase Cyclodex B column without further purification, using the conditions described above.

### Study site

We selected a site in Utah where adult VLB had been collected during statewide pest surveys in 2013 and during preliminary bioassays in 2014. Field bioassays were conducted at Adams Produce, a commercial orchard in Pleasant Grove (Utah County). The orchard was planted with mature cherry and peach trees (position of first trap in first replicate 40.3622, −111.7047, 1562 m elevation), and surrounded by residential areas.

### Field bioassays

Black intercept panel traps (Alpha Scents Inc., West Linn, OR) coated with Fluon® PTFE dispersion by the trap manufacturer were used in all field assays. Traps were suspended from 1.5 m tall L-shaped hangers constructed from 2.5 cm diam PVC irrigation pipe, which were mounted on 2.5 cm × 1.2 m sections of steel reinforcing bar driven partway into the ground. Trap collection cups were filled with ~200–400 ml propylene glycol, which acted as a surfactant and preservative. Traps were positioned ~10 m apart in approximately linear transects.

Ultra-high release rate (UHR) ethanol lures were used according to manufacturer instructions (release rate ~0.5 g/d; Alpha Scents, West Linn, OR). UHR ethanol lures were replaced when dry (generally after ~45 d). Candidate attractant solutions and solvent controls were dispensed into clear plastic sachets (2 mil wall thickness, 5 × 7.5 cm press-seal bags), sealed, and suspended with wire or plastic cable ties in the center open area of the trap. Candidate attractant lures contained 25 mg of each stereoisomer being tested, diluted in 1 ml of isopropyl alcohol (solvent). Solvent controls contained 1 ml of isopropyl alcohol. Treatments and controls were replaced weekly. Blank traps contained dry sachets or no lures.

Treatments were randomly assigned to traps within each five-trap replicate, and each trap was rotated one position along the transect each week to minimize positional effects. Whole traps, rather than treatments, were rotated to avoid any residual effects of other lures. Traps were checked weekly, at which time the trapped insects were removed and preserved in 70% ethanol until sorted and identified.

We tested responses of adult VLB to traps baited with trichoferone in two bioassays during 2016. To time the experiments for the beetles’ flight period, we deployed sentinel traps baited with UHR ethanol from 11 May until 21 June 2016. The first adult beetles were captured on 14 June.

During the first experiment (21 June until 19 July), we tested (1) the synthetic blend of all four stereoisomers of trichoferone, (2) a synthetic blend containing the insect-produced (2*R*,4*R*)- and (2*R*,4*S*)-isomers (~1:1 ratio), (3) UHR ethanol, (4) isopropanol solvent control, and (5) blank control traps. The second experiment tested whether ethanol, which is known to enhance attraction to pheromone lures for some other cerambycids^47^, might increase attraction of VLB. Lures consisted of (1) the four stereoisomer blend, (2) the four stereoisomer blend + UHR ethanol, (3) UHR ethanol alone, (4) isopropanol solvent control, and (5) blank controls. The experiment was deployed from 19 July until 2 August. Both bioassays were conducted at the Adams Produce site, and 10 replicates were deployed for each study.

### Data analysis

Captured beetles were sexed using the sexually dimorphic characters of pronotum width and margin of abdominal sternite IV^33^. Trap catch data were analyzed using Dell Statistica 64 Academic software (Version 13^47^). Differences between treatments in mean numbers of beetles captured per trap per week were tested with the non-parametric Friedman ANOVA because assumptions of parametric ANOVA were violated by unequal variance. We tested for differences between pairs of treatment means using a post-hoc means separation test for Friedman ANOVA^48^. Bias in sex ratio was examined using a X^2^-test, assuming 1:1 sex ratio in natural beetle populations.

Specimens of VLB are retained at the USDA Otis Laboratory (Buzzards Bay, MA), and the Pennsylvania Department of Agriculture. Cerambycid bycatch are maintained at the Pennsylvania Department of Agriculture. Voucher specimens of VLB have been submitted to the Entomological Research Collection at the University of California, Riverside (Riverside, CA) (Voucher #s UCRC ENT 511203-511210).

## Supplementary information

Supplement

## Data Availability

Data are the property of the U.S. Department of Agriculture, are maintained on U.S. government computers, and are of public record. Bioassay data are available at www.exhibit.xavier.edu/biology_faculty/107. Specimens of VLB that are retained by the authors and the Pennsylvania Department of Agriculture are available by request. Figure 1 was assembled using Adobe Photoshop CS3 & CS5; images were cropped to a uniform size and contrast/brightness was adjusted, but otherwise remain unaltered.
