# Supplementary material for: Isolation and identification of a male-produced aggregation-sex pheromone for the velvet longhorned beetle, Trichoferus campestris
Source: Sci Rep. 2019 Mar 14;9:4459. doi: 10.1038/s41598-019-41047-x (PMC6418187; doi:10.1038/s41598-019-41047-x)

ONLINE SUPPLEMENT:

Isolation and identification of a male-produced aggregation-sex pheromone for the velvet longhorned beetle, *Trichoferus campestris*

Ann M. Ray[1]*, Joseph A. Francese[2], Yunfan Zou[3], Kristopher Watson[4], Damon J. Crook[2], Jocelyn G. Millar[3]

[1]Department of Biology, Xavier University, 1548 Musketeer Dr., Cincinnati, OH 45207, USA

[2]USDA-APHIS-PPQ-CPHST Otis Laboratory, 1398 W. Truck Rd., Buzzards Bay, MA 02542, USA

[3]Department of Entomology, University of California, 900 University Avenue, Riverside, CA 92521, USA

[4]Utah Department of Agriculture and Food, 350 North Redwood Road, PO Box 146500, Salt Lake City, Utah 84114, USA

*Corresponding author

E-mail: raya6@xavier.edu

Supplementary Table S1: Trapping details, total trap catch, and number of individuals of each sex captured in all traps during field bioassays at Adams Produce in 2014-2015 (position of first trap in first replicate/s 40.3622, -111.7047, 1562 m elevation). Asterisks denote that lures contained a 1:1 racemic blend of enantiomers. Two asterisks indicate that significantly more females were captured than males across all treatments (assuming 1:1 sex ratio in the population, X^2-test, d.f.=1, p <0.00001 for each test).

**Dates**	**Treatments (in each replicate)**	**# replicates**	**Total VLB**	**# males**	**# female**
16 VII—7 VIII 2014	3*R**-hydroxy decan-2-one, Oct-3-en-3-one, *syn*-2,3 octanediol (RR), *anti*-2,3 octanediol (RS), 3*R**-hydroxy octan-2-one, *syn*-2,3 hexanediol (RR), *anti*- 2,3 hexanediol (RS), 3*R**-hydroxy hexan-2-one, 3*R**-hydroxy butan-2-one [acetoin], Ultra High Release [UHR] ethanol, blank	3	104	11	87**
10 VI—18 VIII 2015	UHR ethanol, UHR ethanol + acetoin, acetoin, solvent control, blank	10	731	142	589**

Supplementary Figure S1: Additional images of sections of thoraces of adult male (a-c: 40X magnification) and female (d: 63X magnification) *T. campestris* showing presence of subcuticular glands connected to pores in males. Glands and pores are absent from the prothoraces of females.

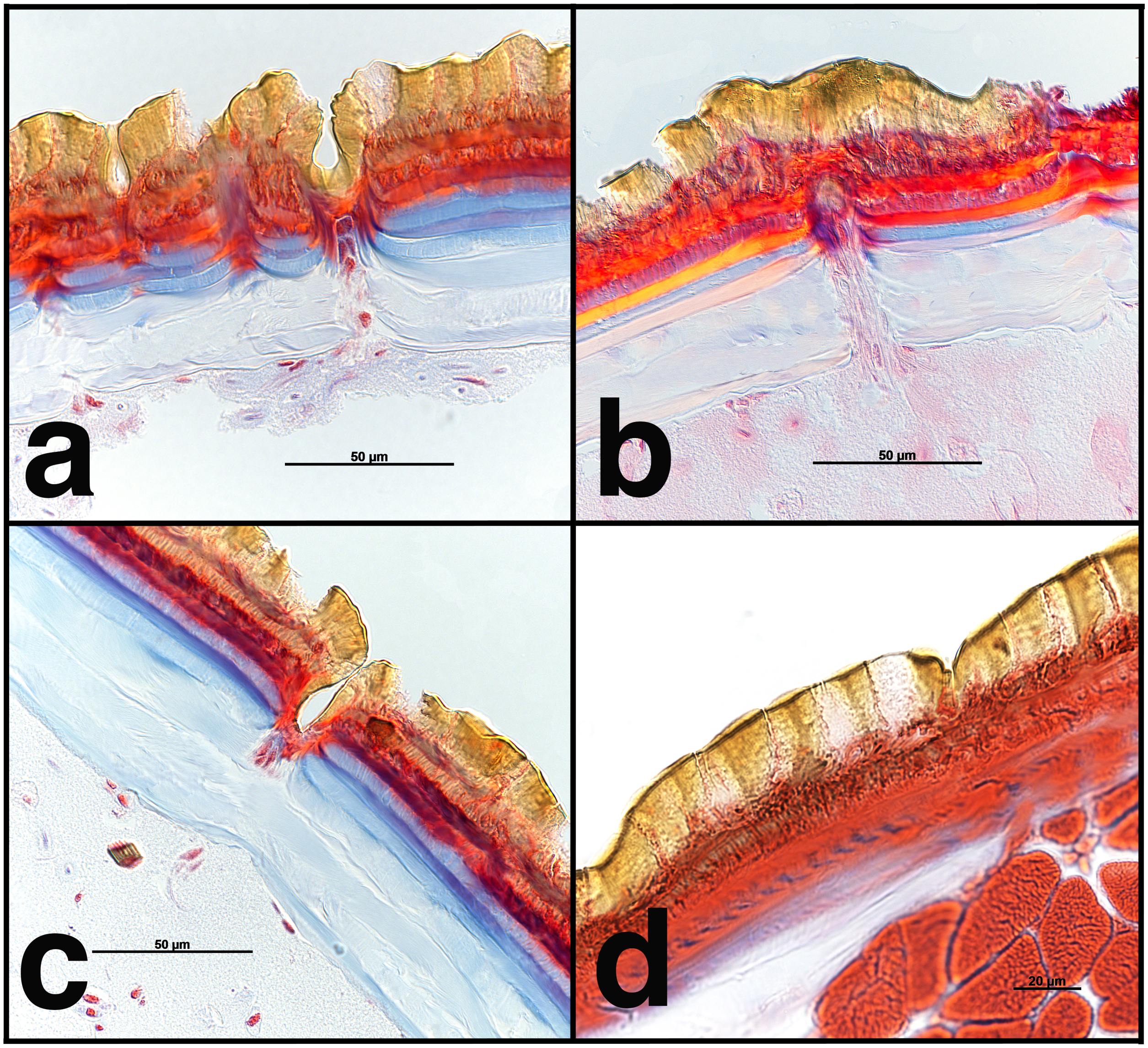

Supplementary Figure S2: Results of 2014 bioassays, showing mean number of adult VLB captured per trap per week. Error bars indicate the standard error of the mean. N=15, X^2 ANOVA=15.01, d. f. = 10, p=0.1317, differences between treatment means are n.s. at $\alpha \leq 0.05$

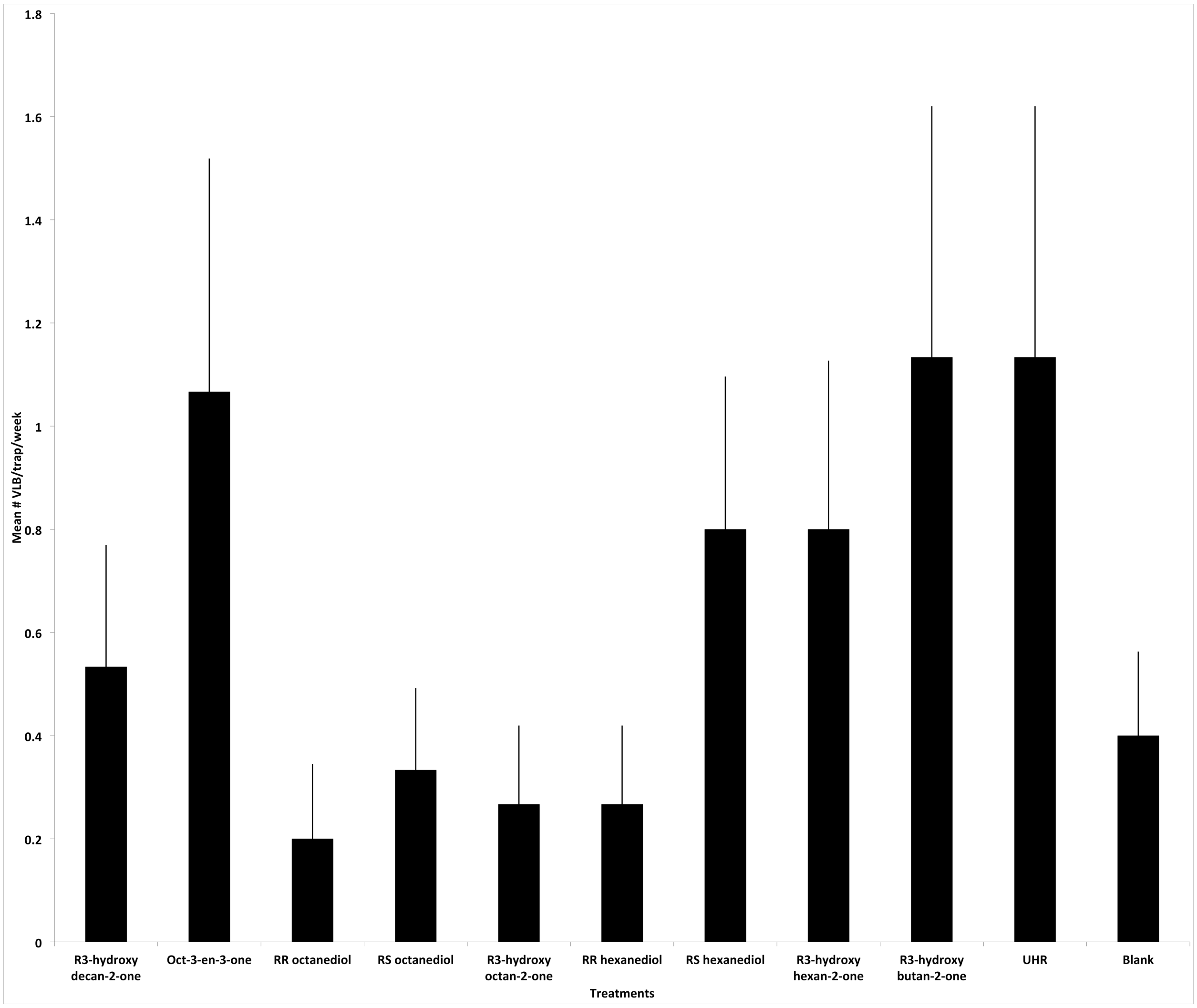

Supplementary Figure S3: Results of 2015 bioassays, showing mean number of adult VLB captured per trap per week. Error bars indicate the standard error of the mean. Significantly more adult beetles were captured in traps baited with UHR ethanol than in acetoin-only or solvent-only traps, however there was no significant difference between mean number of beetles captured in UHR ethanol versus blank control; N=100, d.f.=4 X^2 ANOVA=37.525, d.f.=4, p=2.127 x 10^{-61}, treatment means with the same letters are not significantly different at α=0.05.

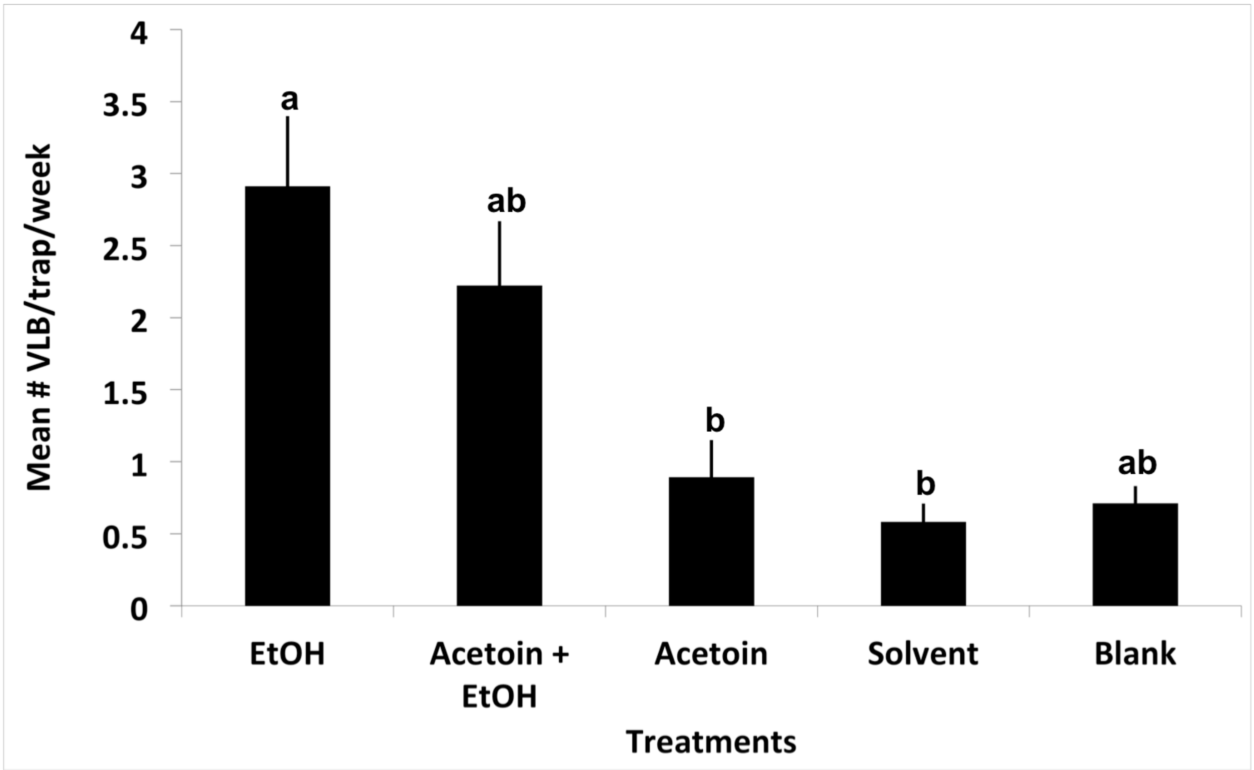

Supplement: Supplementary file 1 — Supplement [file 41598_2019_41047_MOESM1_ESM.pdf]
